# Uncovering Male Fertility Transition Responsive miRNA in a Wheat Photo-Thermosensitive Genic Male Sterile Line by Deep Sequencing and Degradome Analysis

**DOI:** 10.3389/fpls.2017.01370

**Published:** 2017-08-08

**Authors:** Jian-Fang Bai, Yu-Kun Wang, Peng Wang, Wen-Jing Duan, Shao-Hua Yuan, Hui Sun, Guo-Liang Yuan, Jing-Xiu Ma, Na Wang, Feng-Ting Zhang, Li-Ping Zhang, Chang-Ping Zhao

**Affiliations:** ^1^Beijing Engineering and Technique Research Center for Hybrid Wheat, Beijing Academy of Agriculture and Forestry Sciences Beijing, China; ^2^The Municipal Key Laboratory of Molecular Genetic of Hybrid Wheat, Beijing Academy of Agriculture and Forestry Sciences Beijing, China; ^3^College of Plant Science and Technology, Beijing University of Agriculture Beijing, China; ^4^College of Life Science, Capital Normal University Beijing, China

**Keywords:** *Triticum aestivum* L., photoperiod thermo-sensitive genic male sterility (PTGMS), miRNA, fertile transformation, RNA-seq

## Abstract

MicroRNAs (miRNAs) are endogenous small RNAs which play important negative regulatory roles at both the transcriptional and post-transcriptional levels in plants. Wheat is the most commonly cultivated plant species worldwide. In this study, RNA-seq analysis was used to examine the expression profiles of miRNA in the spikelets of photo-thermosenisitive genic male sterile (PTGMS) wheat line BS366 during male fertility transition. Through mapping on their corresponding precursors, 917–7,762 novel miRNAs were found in six libraries. Six novel miRNAs were selected for examination of their secondary structures and confirmation by stem-loop RT-PCR. In a differential expression analysis, 20, 22, and 58 known miRNAs exhibited significant differential expression between developmental stages 1 (secondary sporogenous cells had formed), 2 (all cells layers were present and mitosis had ceased), and 3 (meiotic division stage), respectively, of fertile and sterile plants. Some of these differential expressed miRNAs, such as tae-miR156, tae-miR164, tae-miR171, and tae-miR172, were shown to be associated with their targets. These targets were previously reported to be related to pollen development and/or male sterility, indicating that these miRNAs and their targets may be involved in the regulation of male fertility transition in the PTGMS wheat line BS366. Furthermore, target genes of miRNA cleavage sites were validated by degradome sequencing. In this study, a possible signal model for the miRNA-mediated signaling pathway during the process of male fertility transition in the PTGMS wheat line BS366 was developed. This study provides a new perspective for understanding the roles of miRNAs in male fertility in PTGMS lines of wheat.

## Introduction

MicroRNAs (miRNAs) are an extensive class of non-coding small endogenous RNAs approximately 21 nucleotides (nts) in length that are crucial regulators of gene expression in plants (Bartel, 2004). MiRNAs were first described by Lee et al. (1993) in *C. elegans* (Lee et al., 1993). To date, 46,231 mature miRNAs have been identified in the Sanger miRNA database (miRBase, version 21.0). In plants, these miRNAs are usually transcribed into primary miRNAs (pri-miRNAs) by RNA polymerase (Pol II) (Lee et al., 2004). The pri-miRNA is then processed into a stem-loop precursor miRNA (pre-miRNA) and further processed by Dicer-like 1 (DCL1), or occasionally by DCL4, into a mature miRNA duplex (miRNA/miRNA^*^) with 20–22 nt in the cell nucleus (Rajagopalan et al., 2006). Subsequently, the miRNA/miRNA^*^ is exported into the cytoplasm by Hasty (the plant ortholog of exportin 5) (Yang et al., 2006). The miRNA/miRNA^*^ interacts with argonaute (AGO) and is then incorporated into the RNA-induced silencing complex (RISC) which mediates the unwinding of the miRNA/miRNA^*^ (Baumberger and Baulcombe, 2005). The miRNA serves as a guide for recognition of its target transcripts and interacts with its target genes by perfect or near-perfect complementary base pairing, whereas the miRNA^*^ is often degraded (Bartel, 2004; Park et al., 2005; Jung et al., 2009). Finally, RISC suppresses the expression of mRNA targets in a sequence specific manner by degrading target mRNAs or repressing the translation process (Bartel, 2004). Extensive studies have demonstrated that miRNA plays a critical role in many biological processes such as organ senescence (Lim et al., 2010; Pei et al., 2013), flowering time (Wollmann et al., 2010), reproductive development (Peng et al., 2012; Yan et al., 2015), leaf development (Scarpella et al., 2010), fertility transition (Tang Z. et al., 2012; Ding et al., 2016), and root morphology (Moreno-Risueno et al., 2010). miRNAs are also reported to be associated with plant responses to biotic and abiotic stresses (Leung and Sharp, 2010; Sunkar, 2010; Todesco et al., 2010; Tang Z. et al., 2012; Feng et al., 2014; Shu et al., 2016).

Hybrid cultivars have been widely used in plant production to improve yield and have other favorable agronomic traits. For wheat, *Triticum aestivum* L. (2n = 6x = 42), one of the main grain crops for humans, hybrid cytoplasmic male sterility (CMS) systems or environment-sensitive sterility systems are the basis of hybridization systems in China and India (Longin et al., 2012). Currently, cytoplasmic male sterility lines (CMS) with a maintenance line and a restoration line are mainly used in a three-line breeding system, whereas environment (temperature and photoperiod)-sensitive sterility systems (PTGMS) are mainly used in a two-line breeding system. Plant fertility is controlled by temperature and/or photoperiod in environment-sensitive sterility systems. For example, the critical temperature for anther/pollen (male) fertility transition of PTGMS line BS20 is in the range of 10–13°C for temperature and 11.5–12 h for light period, during the spikelet differentiation stage. At the day average temperature of 12°C, the fertility of BS20 increased and the critical temperature decreased with longer day length (Ru et al., 2015). Thus, the PTGMS line can be used as both a maintenance line and a male-sterile line for exploiting heterosis that can improve crop yield, enhance resistance, and improve crop quality (Jordaan, 1996; Virmani and Ilyas-Ahmed, 2001; Yang et al., 2007; Tang et al., 2011; Tang Z. et al., 2012; Zhou et al., 2014; Wang et al., 2017).

To date, many miRNAs have been identified in anther development of male sterile lines, such as maize (Shen et al., 2011), cotton (Wei et al., 2013), *Brassica juncea* (Yang et al., 2013), wheat (Tang Z. et al., 2012), soybean (Ding et al., 2016), tomato (Omidvar et al., 2015), and rice (Yan et al., 2015). Some of these miRNAs may be involved in plant fertility. Overexpression of osa-miR159 caused male sterility in rice, while mutating the target of osa-miR159 also resulted in abnormalities in anther and pollen development (Kaneko et al., 2004; Tsuji et al., 2006). Recently, osa-miR164d and osa-miR444f were identified and confirmed to be connected to pollen development by interacting with their targets in a rice PTGMS line (Zhang et al., 2016). In *Arabidopsis*, miRNA172, interacting with its target genes APETALA2-like, can control flowering time (Chen, 2004). Moreover, overexpression of miRNA167 can lead to male fertility defects (Siré et al., 2009). Furthermore, some studies have reported that MYB33/MYB65 which could be down-regulated by miRNA159a, accelerates anther development in *Arabidopsis* (Millar and Gubler, 2005).

Tang Z. et al. (2012) found that miR167 and transacting small interfering RNA (tasiRNA)-ARF played roles in cold-induced male sterility in wheat (Tang Z. et al., 2012). However, few studies have demonstrated the relationship between miRNA and male fertility transition in PTGMS wheat lines. The genetic mechanism is complex and the sterility mechanism is still not clear, both of which are obstacles that restrict its application. In this study, we identified conserved and novel miRNAs which may be involved in male fertility transition during pollen and anther development in PTGMS wheat line BS366 using RNA-seq.

## Materials and methods

### Plant materials, growth conditions, and sample collection

The wheat (*Triticum aestivum* L.) PTGMS line BS366 was used in this study. Plants were grown in soil in plastic pots and vernalized naturally in the field. After the four-leaf stage (when the tip of the fourth leaf had emerged), plants were randomly divided into two groups. One group (fertile condition) was transferred to an artificial climate incubator at 20°C (with 12-h day/12-h night) and relative humidity of 60–80% for the entire reproductive period. The other group (sterile condition) was held at 10°C (with 12-h day/12-h night). The plants received cold stress for 5 days. Based on leaf age index and anther length, spikelets were collected from each treatment group at three different developmental stages identified using a dissecting microscope (Olympus SZX12,Tokyo, Japan): stage 1: secondary sporogenous cells had formed (anther length: 1.5 mm); stage 2: all cell layers were present and mitosis had ceased (anther length: 2.2 mm), and stage 3: meiotic division stage (anther length: 3.0 mm) (Tang et al., 2011; Tang Z. et al., 2012). Therefore, samples from these two groups, fertile plants and sterile plants, were designated FS1, FS2, FS3 and SS1, SS2, SS3 for each developmental stage, respectively. Small RNA and degradome sequencing was previously carried out for these six sample sets (Tang Z. et al., 2012) and further analyzed here under Sections Small RNA Sequence Data Analysis. For the current work we also collected anther samples from the same stages.

### Sequence data source

Small RNA sequencing and degradome sequencing data from Tang Z. et al. (2012) were downloaded from Gene Expression Omnibus (http://www.ncbi.nlm.nih.gov/geo/) under GEO accession numbers GSE36867 and GSE37134, respectively. The GO database was downloaded from the Gene Ontology website (http://www.geneontology.org/GO.downloads.shtml) (Ashburner and Bergman, 2005). The KEGG database was obtained from the KEGG website (ftp://ftp.genome.jp/pub/kegg/pathway/) (Kanehisa and Goto, 2000).

### Small RNA sequence data analysis

After filtering the low-quality reads (reads less than 18 nt and reads whose adaptors were null) and removing adaptors/adaptor sequences, clean reads in the range of 18–30 nt were retained for further analysis. rRNAs, tRNAs, small nuclear (sn)RNAs, and small nucleolar (sno)RNAs were identified and eliminated by comparing clean reads to GenBank (http://www.ncbi.nlm.nih.gov) and Rfam (12.0) (http://rfam.xfam.org/) RNA family databases (Gardner et al., 2009). Known miRNAs in the samples were identified by comparison with the specified range in the miRNA database (http://www.mirbase.org). Unannotated small RNA sequences were used for predicting novel miRNAs based on the characteristic hairpin structure of miRNA precursors. Novel miRNAs were predicted using miRdeep2 (https://www.mdcberlin.de/rajewsky/miRDeep). The secondary structures were predicted using the MFOLD3.2 web server (http://mfold.rna.albany.edu/) (Zuker, 2003). Wheat (*Triticum aestivum* L.) transcripts, downloaded from phytozome v11 (www.phytozome.net), were used to determine potential target mRNA candidates for miRNAs using target gene prediction software psRNATarget with default parameters (http://plantgrn.noble.org/psRNATarget/) (Dai and Zhao, 2011) according to the rules proposed by Allen et al. (2005) and Schwab et al. (2005). Functional annotations of predicted targets were analyzed using the Blast2GO program (*P* ≤ 0.05) to obtain the GO annotations from the unigene database using BLASTX hits against the available Nr database in NCBI (Conesa et al., 2005). The KEGG pathway was analyzed using BLASTX (*P* ≤ 0.05) through the KEGG database.

### Degradome sequence data analysis

We used the CleaveLand pipeline to find sliced miRNA targets using wheat unigenes, Viridiplantae miRBase 21.0 mature miRNAs, and miRNAs identified in our study as inputs (Addo-Quaye et al., 2008). To allow for inaccurate target cleavage or variation in miRNA 5′ ends, the pipeline was modified to recognize targets cleaved between the 10th and 11th nucleotides of mature miRNA.

### RNA extraction and qRT-PCR validation of miRNAs and target gene expression

Total RNA was obtained for each anther sample using TRIzol Reagent (Invitrogen, Nottingham, UK) according to the manufacturer's instructions. The purified RNA was dissolved in RNase-free water and stored at −80°C until subsequent analysis.

RNAs isolated from anthers were transcribed into cDNA using M-MLV Reverse Transcriptase according to the manufacturer's instructions (Takara, Japan). Quantitative real-time PCR (qRT-PCR) was performed with a SYBR Premix Ex Taq™ kit (Takara, Japan) on a Roche LightCycler 480 real-time PCR machine, following the manufacturer's instructions. The protocol used to perform qRT-PCRs was 10 min at 95°C, followed by 40 cycles of 15 s at 95°C and 60 s at 61°C. Ubiquitin was used as a reference gene for miRNA targets analysis, and U6 was used as a reference gene for miRNA analysis. The reactions were performed with three biological replicates and at least two technical replicates per sample. The data were analyzed using the 2^−ΔΔCt^ method (Livak and Schmittgen, 2001), and means ± standard errors (SE) of three biological replicates are presented. The primers for the miRNA qRT-PCR are listed in Table S1.

## Results

### Overview of small RNA sequences data

**Figure 1:**
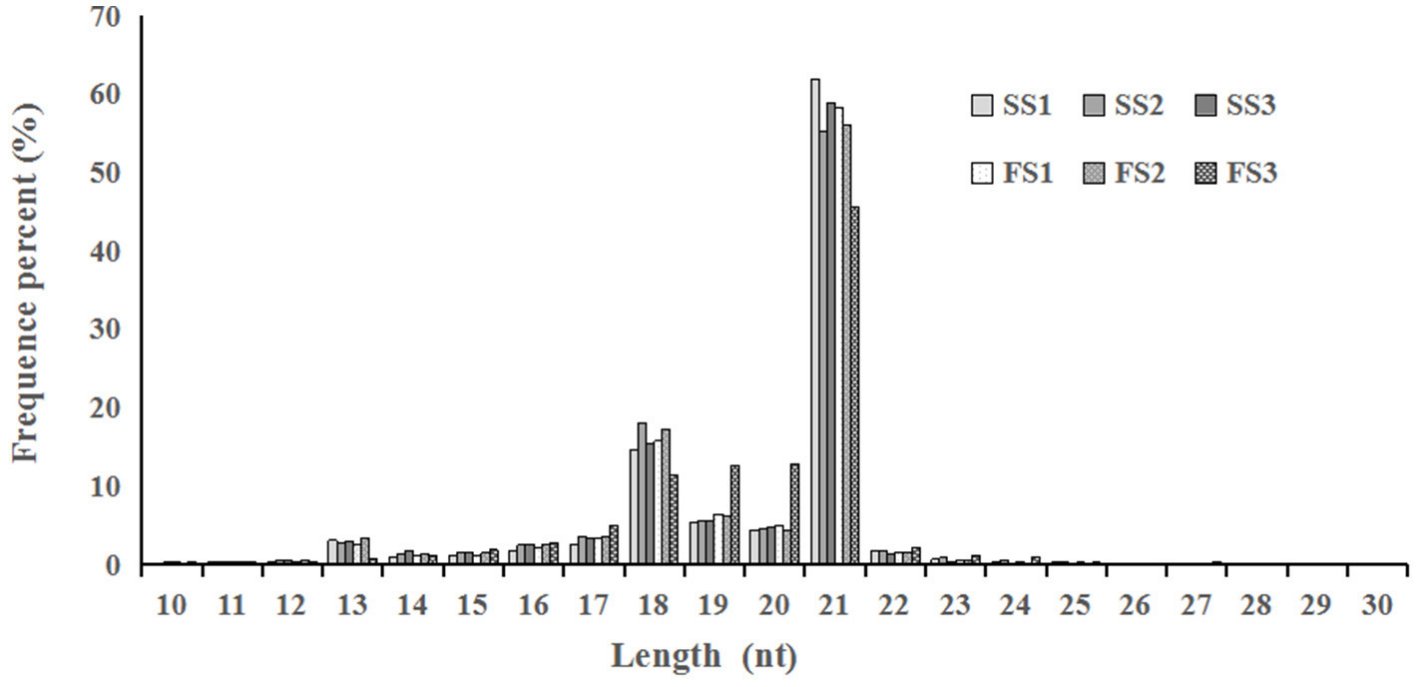
Length distribution and abundance of micro(mi)RNAs in libraries from different pollen developmental stages in two treatments (fertile or sterile) of wheat. In all six libraries, the majority of miRNAs were 18–21 nt in the length, with 21 nt being the most abundant.

Photo-thermosensitive genic male sterile (PTGMS) line wheat had abnormal fertility when treated with low temperature during the meiosis stage (Tang et al., 2011). In this study, the goals were to identify new miRNAs which may be involved in the regulation of wheat male-sterility from a PTGMS wheat line and to illustrate the regulation mechanism of miRNAs in the low temperature–male fertility transition network. Small RNA sequencing data were generated by Solexa technology and yielded 11.4–13.5 million raw reads of each sample from all six small RNA libraries. Approximately 86% were clean reads and were kept for further analysis after eliminating the unavailable sequences (low quality reads, 3′ adapter null, 5′ adapter null, insert null, reads shorter than 18 nt, and polyA reads) (Table 1). The most abundant size classes of sRNA ranged from 18 to 21 nt, and the most abundant size sRNA in all six libraries was 21 nt (45.56–61.89%) (Figure 1). Thus, the sRNA transcriptome in wheat is dominated by 21 nt sRNAs. This is in contrast with other plant species, including *Arabidopsis* (Fahlgren et al., 2007), rice (Sunkar et al., 2008), peanut (Chi et al., 2011), radish (Xu et al., 2013), and cucumber (Martínez et al., 2011; Mao et al., 2012), where 24 nt sRNAs are more abundant.

**Table 1:** Statistics of small RNA sequencing data of PTGMS line BS366 under sterile (SS) and fertile condition (FS).

**Sample^a^**	**Total reads**	**High quality**	**Smaller than 18 nt**	**Adaptors**	**Insert null**	**PloyA**	**Clean reads**
SS1	11,770,854	11,540,935	1,195,377	114,129	488	1,531	10,229,410 (88.64%)
SS2	13,151,774	12,891,271	1,637,353	113,403	489	2,159	11,137,867 (86.40%)
SS3	11,989,719	11,680,714	1,531,187	93,847	526	2,448	10,052,706 (86.06%)
FS1	13,474,821	13,234,198	1,525,764	116,539	526	2,900	11,588,469 (87.56%)
FS2	11,434,861	11,232,668	1,495,943	98,328	553	1,512	9,636,332 (85.79%)
FS3	12,592,817	12,264,818	1,502,243	178,986	455	3,459	10,579,675 (86.26%)

a *Total of six small RNA cDNA libraries derived from PTGMS plants in sterile (SS) and fertile condition (FS) of different stage, respectively. The number 1, 2, 3 indicated stage 1: secondary sporogenous cells had formed; stage 2: all cells layers were present and mitosis had ceased, and stage 3: meiotic division stage, respectively*.

The clean small RNAs reads were mapped to the wheat genome in order to analyze their expression and distribution in the genome. The total and unique sRNAs were classified into different RNA categories (Table 2). More than approximately 91% of unique small RNAs corresponding to more than 92% of total small RNAs from the different libraries (except for FS3 25.13%/35.98%) were mapped onto the wheat genome (Table 2). After removing non-coding RNAs including rRNA, tRNA, snRNA, and snoRNA by blasting sRNA sequences against Genbank and Rfam, we annotated and obtained 2378 (0.06%), 2627 (0.06%), 2655 (0.06%), 2733 (0.06%), 2770 (0.06%), 2461 (0.06%), and 713 (0.01%) unique reads in SS1, SS2, SS3, FS1, FS2, and FS3, respectively. These unique reads were similar to known miRNAs.

**Table 2:** Distribution of small RNAs among different categories in different development stage of PTGMS line BS366 under different condition.

**Category**	**SS1**				**SS2**				**SS3**				**FS1**				**FS2**				**FS3**			
	**Unique sRNAs (%)**		**Total sRNAs (%)**		**Unique sRNAs (%)**		**Total sRNAs (%)**		**Unique sRNAs (%)**		**Total sRNAs (%)**		**Unique sRNAs (%)**		**Total sRNAs (%)**		**Unique sRNAs (%)**		**Total sRNAs (%)**		**Unique sRNAs (%)**		**Total sRNAs (%)**	
Total	4,234,853	100.00	10,229,410	100.00	4,286,995	100.00	11,137,867	100.00	4,468,812	100.00	10,052,706	100.00	4,671,289	100.00	11,588,469	100.00	4,037,358	100.00	9,636,332	100.00	5,057,900	100.00	10,579,675	100.00
Match_genome	3,855,146	91.03	9,407,748	91.97	3,917,068	91.37	10,334,127	92.78	4,082,040	91.35	9,268,237	92.20	4,271,167	91.43	10,690,868	92.25	3,688,383	91.36	8,901,224	92.37	1,271,126	25.13	4,019,074	37.99
Exon_antisense	17,536	0.41	41,963	0.41	18,464	0.43	47,312	0.42	19,928	0.45	49,006	0.49	19,342	0.41	49,467	0.43	17,464	0.43	40,902	0.42	11,696	0.23	24,146	0.23
Exon_sense	48,708	1.15	80,220	0.78	64,153	1.50	101,089	0.91	39,463	0.88	81,722	0.81	53,670	1.15	94,512	0.82	43,623	1.08	75,077	0.78	16,393	0.32	35,014	0.33
Intron_antisense	24,359	0.58	46,505	0.45	25,129	0.59	49,305	0.44	26,028	0.58	47,436	0.47	27,572	0.59	54,170	0.47	23,179	0.57	42,676	0.44	20,356	0.40	45,249	0.43
Intron_sense	900,590	21.27	2,062,146	20.16	912,889	21.29	2,231,617	20.04	956,376	21.40	2,046,554	20.36	996,022	21.32	2,340,279	20.19	859,361	21.29	1,921,980	19.95	307,660	6.08	946,856	8.95
miRNA	2,378	0.06	493,139	4.82	2,627	0.06	563,412	5.06	2,723	0.06	579,600	5.77	2,770	0.06	604,988	5.22	2,461	0.06	597,705	6.20	713	0.01	421,921	3.99
rRNA	37,932	0.90	521,297	5.10	47,310	1.10	815,699	7.32	31,402	0.70	341,584	3.40	41,964	0.90	652,531	5.63	34,836	0.86	507,004	5.26	43,217	0.85	672,965	6.36
snRNA	817	0.02	4,045	0.04	958	0.02	4,072	0.04	746	0.02	3,006	0.03	917	0.02	5,047	0.04	770	0.02	3,529	0.04	198	0.00	574	0.01
snoRNA	341	0.01	766	0.01	361	0.01	868	0.01	200	0.00	362	0.00	314	0.01	751	0.01	247	0.01	605	0.01	120	0.00	577	0.01
tRNA	1,654	0.04	15,613	0.15	2,037	0.05	18,149	0.16	1,375	0.03	9,832	0.10	1,803	0.04	16,646	0.14	1,511	0.04	12,410	0.13	1,809	0.04	17,441	0.16
Unannotion	3,200,538	75.58	6,963,716	68.08	3,213,067	74.95	7,306,344	65.60	3,390,571	75.87	6,893,604	68.57	3,526,915	75.50	7,770,078	67.05	3,053,906	75.64	6,434,444	66.77	4,655,738	92.05	8,414,932	79.54

### Identification of known miRNAs

**Figure 2:**
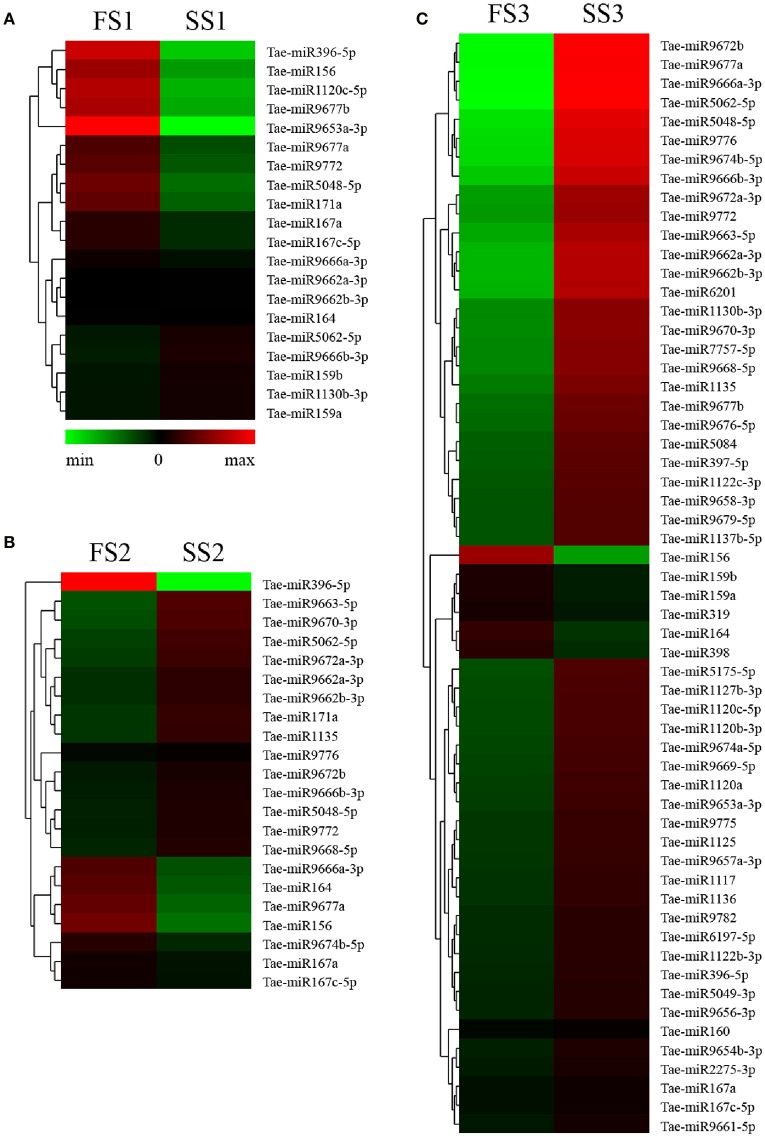
Differential expression analyses of known miRNAs in FS1/SS1 **(A)**, FS2/SS2 **(B)**, and FS3/SS3 **(C)** using RNA-Sequencing.

To identify known miRNAs from three pollen developmental stages, the small RNAs mapped on the genome were analyzed with a homology-based method of mapping the unique sequences using miRBase Release 21.0 (July 3, 2014). Currently, approximately 5554 miRNAs derived from 6992 pre-miRNAs are published in miRBase. In our study, a total of 3304 known miRNAs in the spikes of BS366 at the different developmental stages evaluated (1736 in SS1, 1705 in SS2, 1822 in SS3, 1677 in FS1, 1699 in FS2, and 1980 in FS3) were identified as orthologs of known miRNAs in other species (Table 3), of which 108 miRNAs were from wheat (Table S2). To investigate the regulation of miRNA during male fertility transition in wheat, we compared the expression of miRNAs at different stages of pollen development between fertile plants and sterile plants. The results revealed that there were 20, 22, and 58 differentially expressed miRNAs in FS1/SS1, FS2/SS2, and FS3/SS3, respectively (Table 4, Figure 2). Among these miRNAs, 9 miRNAs in SS1/FS1, 14 miRNAs in SS2/FS2, and 49 miRNAs in SS3/FS3 were up-regulated, and the others were down-regulated (Figure 2). Not all differentially expressed miRNAs expressed at different stage. For example, tae-miR171a showed differential expression in FS1/SS1 and FS2/SS2, not in FS3/SS3. However, many differentially expressed miRNAs such as tae-miR167a, tae-miR156 and tae-miR164, showed differentially expressed miRNAs peaked at stage 3 (Figure 2), suggesting that the transition of male fertility may occur at this time. Furthermore, these miRNAs different in abundance at different stages and conditions. For example, tae-miR167, tae-miR5048, tae-miR5062, tae-miR9666, and tae-miR9672 were found over 10,000 times (Table 4). Remarkably, few or no copies of all known miRNAs from wheat were found in the FS3 library (Table 4 and Table S2), suggesting that expression levels varied markedly among different miRNA families, probably owing to developmental stage-specific expression. This result also indicates that stage 3 (meiotic division) is perhaps the important phase for male fertility transition. In plants, the determination of which strand of the miRNA:miRNA^*^ duplex is incorporated into the RNA induced silencing complex (RISC) is largely based on the identity of the first nucleotide. In *Arabidopsis*, the AGO family consists of 10 members. AGO1 tends to recruit miRNAs beginning with a uracil (U), while AGO2 and AGO4 display preferences for miRNAs with a 5′ terminal adenosine (A), and AGO5 recruits miRNAs initiated with a 5′ cytosine (C) (Mi et al., 2008). In this study, the base preference of 21 nt known miRNAs was analyzed. The results revealed that the order of the bias for the first nucleotide of 21 nt known miRNA is A>G>U>C in SS1, SS2, SS3, FS1, and FS2 (Figure S1). Notably, in FS3, the bias for the first nucleotide of 21 nt miRNA is usually A, suggesting that miRNAs of different stages and different conditions might be recruited by different AGO proteins and enter distinct regulatory pathways.

**Table 3:** Summary of known miRNAs in each sample.

	**miRNA**	miRNA^*^	**miRNA-5p**	**miRNA-3p**	**miRNA precursors**	**Unique sRNAs matched to miRNA precursors**	**Total sRNAs matched to miRNA precursors**
Known miRNA in miRbase	5,554	0	1,466	1,430	6,992	–	–
SS1	1,736	0	430	354	2,348	2,455	493,494
SS2	1,705	0	439	344	2,321	2,717	564,215
SS3	1,822	0	428	373	2,454	2,791	579,988
FS1	1,677	0	418	344	2,276	2,867	605,744
FS2	1,699	0	423	339	2,305	2,536	598,192
FS3	1,980	0	401	302	2,603	794	422,385

**Table 4:** List of differential expressed wheat miRNAs between different decelopment stage in sterile condition (SS) and fertile condition (FS) in this study.

**List of differential expressed wheat miRNAs between FS1 and SS1**								
**miR_name**	**FS1-reads**	**SS1-reads**	**log2Ratio(SS1/FS1) normalized**	***p*****-value**	***q*****-value (Storey and Tibshirani**, **2003****)**	**UP_Down**	**Sequences**	**Length**
tae-miR5062-5p	18,519	21,541	0.551065615	0	0	Up	UGAACCUUAGGGAACAGCCGCAU	23
tae-miR9677a	49,526	30,779	−0.353253159	1.40E-299	2.32E-298	Down	UGGCCGUUGGUAGAGUAGGAGA	22
tae-miR9666b-3p	3,630	4,316	0.582711903	1.14E-73	1.25E-72	Up	CGGUUGGGCUGUAUGAUGGCGA	22
tae-miR5048-5p	4,408	2,264	−0.628263679	2.71E-68	2.25E-67	Down	UUUGCAGGUUUUAGGUCUAAGU	22
tae-miR9666a-3p	26,852	24,473	0.199148003	8.50E-61	5.63E-60	Up	CGGUAGGGCUGUAUGAUGGCGA	22
tae-miR9662a-3p	3,661	3,623	0.317933618	2.16E-21	1.02E-20	Up	UUGAACAUCCCAGAGCCACCG	21
tae-miR9662b-3p	3,661	3,623	0.317933618	2.16E-21	1.02E-20	Up	UGAACAUCCCAGAGCCACCGG	21
tae-miR1130b-3p	625	709	0.514916024	6.12E-11	2.53E-10	Up	UCUUAUAUUAUGGGACGGAGG	21
tae-miR156	249	96	−1.042052845	1.85E-10	6.80E-10	Down	UGACAGAAGAGAGUGAGCACA	21
tae-miR9772	1,113	651	−0.440737558	2.40E-10	7.96E-10	Down	UGAGAUGAGAUUACCCCAUAC	21
tae-miR167a	67,908	52,304	−0.043673675	3.47E-09	1.04E-08	Down	UGAAGCUGCCAGCAUGAUCUA	21
tae-miR171a	678	372	−0.532996066	4.13E-09	1.14E-08	Down	UGAUUGAGCCGUGCCAAUAUC	21
tae-miR1120c-5p	147	49	−1.251975914	1.41E-08	3.58E-08	Down	UAAUAUAAGAACGUUUUUGAC	21
tae-miR167c-5p	68,035	52,499	−0.04100059	2.76E-08	6.53E-08	Down	UGAAGCUGCCAGCAUGAUCUGC	22
tae-miR164	774	779	0.342276348	2.64E-06	5.83E-06	Up	UGGAGAAGCAGGGCACGUGCA	21
tae-miR396-5p	66	19	−1.46348002	1.84E-05	3.82E-05	Down	AACUGUGAACUCGCGGGGAUG	21
tae-miR9677b	74	26	−1.176027061	0.000127882	0.000249069	Down	CAGGGCGGGGAACAGGUGGCC	21
tae-miR9653a-3p	34	7	−1.947121333	0.000169241	0.00031131	Down	UUUGAGACUUUGGCCAUGGCC	21
tae-miR159b	179	204	0.521596151	0.000389703	0.000679112	Up	UUUGGAUUGAAGGGAGCUCUG	21
tae-miR159a	179	203	0.514506726	0.000475035	0.000786424	Up	UUUGGAUUGAAGGGAGCUCUG	21
**List of differential expressed wheat miRNAs between FS2 and SS2**.								
**miR_name**	**FS2-reads**	**SS2-reads**	**log2Ratio(SS2/FS2) normalized**	***p*****-value**	***q*****-value (Storey and Tibshirani**, **2003****)**	**UP_Down**	**Sequences**	**Length**
tae-miR9677a	37,359	20,970	−0.717174926	0	0	Down	UGGCCGUUGGUAGAGUAGGAGA	22
tae-miR9666a-3p	35,543	22,469	−0.545675488	0	0	Down	CGGUAGGGCUGUAUGAUGGCGA	22
tae-miR5062-5p	16,240	23,628	0.656899936	0	0	Up	UGAACCUUAGGGAACAGCCGCAU	23
tae-miR9672b	45,656	53,013	0.331495768	0	0	Up	UACCACGACUGUCAUUAAGCA	21
tae-miR9662a-3p	3,721	4,830	0.49228685	1.78E-56	2.46E-55	Up	UUGAACAUCCCAGAGCCACCG	21
tae-miR9662b-3p	3,721	4,830	0.49228685	1.78E-56	2.46E-55	Up	UGAACAUCCCAGAGCCACCGG	21
tae-miR9663-5p	961	1,547	0.802818912	3.60E-43	4.27E-42	Up	AAGCGUAGUCGAACGAAUCUG	21
tae-miR9666b-3p	4,755	5,616	0.356051646	9.14E-37	9.48E-36	Up	CGGUUGGGCUGUAUGAUGGCGA	22
tae-miR9672a-3p	1,209	1,710	0.616136132	1.57E-30	1.45E-29	Up	CCACGACUGUCAUUAAGCAUC	21
tae-miR5048-5p	3,052	3,677	0.384728266	4.59E-28	3.81E-27	Up	UUUGCAGGUUUUAGGUCUAAGU	22
tae-miR9670-3p	547	855	0.760337639	1.65E-22	1.25E-21	Up	AGGUGGAAUACUUGAAGAAGA	21
tae-miR164	874	526	−0.616616429	3.22E-15	2.23E-14	Down	UGGAGAAGCAGGGCACGUGCA	21
tae-miR9674b-5p	5,067	4,037	−0.21189419	1.87E-12	1.15E-11	Down	AUAGCAUCAUCCAUCCUACCC	21
tae-miR171a	549	750	0.566038498	1.94E-12	1.15E-11	Up	UGAUUGAGCCGUGCCAAUAUC	21
tae-miR396-5p	69	16	−1.992570405	1.78E-08	9.85E-08	Down	AACUGUGAACUCGCGGGGAUG	21
tae-miR9772	854	1,020	0.372215229	2.34E-08	1.21E-07	Up	UGAGAUGAGAUUACCCCAUAC	21
tae-miR1135	312	423	0.555065686	2.09E-07	1.02E-06	Up	CUGCGACAAGUAAUUCCGAACGGA	24
tae-miR9776	2,996	3,147	0.186893606	3.30E-07	1.52E-06	Up	UUGGACGAGGAUGUGCAACUG	21
tae-miR9668-5p	554	679	0.40947965	6.43E-07	2.81E-06	Up	CCAAUGACAAGUAUUUUCGGA	21
tae-miR167a	62,972	56,678	−0.035967586	1.22E-06	5.08E-06	Down	UGAAGCUGCCAGCAUGAUCUA	21
tae-miR156	195	101	−0.833164779	1.29E-06	5.11E-06	Down	UGACAGAAGAGAGUGAGCACA	21
tae-miR167c-5p	63,072	56,855	−0.0337584	5.10E-06	1.93E-05	Down	UGAAGCUGCCAGCAUGAUCUGC	22
**List of differential expressed wheat miRNAs between FS3 and SS3**								
**miR_name**	**FS3-reads**	**SS3-reads**	**log2Ratio(SS3/FS3) normalized**	***p*****-value**	***q*****-value (Storey and Tibshirani**, **2003****)**	**UP_Down**	**Sequences**	**Length**
tae-miR156	23,134	179	−8.522028941	0	0	Down	UGACAGAAGAGAGUGAGCACA	21
tae-miR9672b	20	50,329	9.789056616	0	0	Up	UACCACGACUGUCAUUAAGCA	21
tae-miR9677a	12	297,26	9.766357621	0	0	Up	UGGCCGUUGGUAGAGUAGGAGA	22
tae-miR9666a-3p	10	26,563	9.867084776	0	0	Up	CGGUAGGGCUGUAUGAUGGCGA	22
tae-miR167a	34,188	56,070	−0.794378559	0	0	Down	UGAAGCUGCCAGCAUGAUCUA	21
tae-miR167c-5p	34,240	56,229	−0.792485913	0	0	Down	UGAAGCUGCCAGCAUGAUCUGC	22
tae-miR5062-5p	7	19,573	9.941104754	0	0	Up	UGAACCUUAGGGAACAGCCGCAU	23
tae-miR164	3,864	779	−3.818517462	0	0	Down	UGGAGAAGCAGGGCACGUGCA	21
tae-miR5048-5p	4	4,666	8.679852991	0	0	Up	UUUGCAGGUUUUAGGUCUAAGU	22
tae-miR9776	4	3,667	8.332266949	0	0	Up	UUGGACGAGGAUGUGCAACUG	21
tae-miR9674b-5p	4	3,415	8.229552262	0	0	Up	AUAGCAUCAUCCAUCCUACCC	21
tae-miR9666b-3p	0	5,158	11.82447846	0	0	Up	CGGUUGGGCUGUAUGAUGGCGA	22
tae-miR9662a-3p	1	2,934	10.01053555	0	0	Up	UUGAACAUCCCAGAGCCACCG	21
tae-miR9662b-3p	1	2,934	10.01053555	0	0	Up	UGAACAUCCCAGAGCCACCGG	21
tae-miR9663-5p	0	1,938	10.41223525	5.44E-203	2.68E-202	Up	AAGCGUAGUCGAACGAAUCUG	21
tae-miR9672a-3p	0	1,395	9.937931805	2.37E-157	1.09E-156	Up	CCACGACUGUCAUUAAGCAUC	21
tae-miR9772	1	1,182	8.698896719	5.88E-157	2.56E-156	Up	UGAGAUGAGAUUACCCCAUAC	21
tae-miR1130b-3p	1	721	7.985737848	5.52E-104	2.27E-103	Up	UCUUAUAUUAUGGGACGGAGG	21
tae-miR7757-5p	1	656	7.849434403	5.57E-96	2.17E-95	Up	AUAAAACCUUCAGCUAUCCAUC	22
tae-miR9670-3p	0	737	9.017403208	5.92E-95	2.19E-94	Up	AGGUGGAAUACUUGAAGAAGA	21
tae-miR9668-5p	0	653	8.84282158	3.92E-86	1.38E-85	Up	CCAAUGACAAGUAUUUUCGGA	21
tae-miR6201	2	535	6.55527748	1.05E-85	3.53E-85	Up	UGACCCUGAGGCACUCAUACCG	22
tae-miR159b	256	104	−2.807677883	1.19E-78	3.76E-78	Down	UUUGGAUUGAAGGGAGCUCUG	21
tae-miR159a	256	104	−2.807677883	1.19E-78	3.76E-78	Down	UUUGGAUUGAAGGGAGCUCUG	21
tae-miR1135	0	457	8.327932754	2.17E-64	6.41E-64	Up	CUGCGACAAGUAAUUCCGAACGGA	24
tae-miR9677b	0	299	7.715884073	2.94E-45	8.37E-45	Up	CAGGGCGGGGAACAGGUGGCC	21
tae-miR9676-5p	0	261	7.519788395	2.33E-40	6.39E-40	Up	UGGAUGUCAUCGUGGCCGUACA	22
tae-miR5084	0	186	7.03104121	3.13E-30	8.27E-30	Up	AUACAGUACUGCAGAGGAUCCUAA	24
tae-miR397-5p	0	178	6.96761583	4.15E-29	1.06E-28	Up	UCACCGGCGCUGCACACAAUG	21
tae-miR1122c-3p	0	150	6.720701089	4.22E-25	1.04E-24	Up	UCUAAUAUUAUGGGACGGAGG	21
tae-miR9658-3p	0	137	6.589914482	3.40E-23	8.11E-23	Up	AUCGUUCUGGGUGAAUAGGCC	21
tae-miR9679-5p	1	133	5.547164834	6.37E-23	1.47E-22	Up	CAGAACCAGAAUGAGUAGCUC	21
tae-miR1137b-5p	0	133	6.547164834	1.33E-22	2.98E-22	Up	UCCGUUCCAGAAUAGAUGACC	21
tae-miR5175-5p	0	113	6.312061361	1.36E-19	2.96E-19	Up	UUCCAAAUUACUCGUCGUGGU	21
tae-miR1127b-3p	0	104	6.192322117	3.29E-18	6.95E-18	Up	ACAAGUAUUUCUGGACGGAGG	21
tae-miR160	159	193	−1.228543519	1.70E-17	3.49E-17	Down	UGCCUGGCUCCCUGUAUGCCA	21
tae-miR1120c-5p	0	98	6.106592243	2.81E-17	5.62E-17	Up	UAAUAUAAGAACGUUUUUGAC	21
tae-miR1120b-3p	0	97	6.091795241	4.03E-17	7.85E-17	Up	UUCUUAUAUUGUGGGACAGAG	21
tae-miR9674a-5p	0	87	5.934825895	1.52E-15	2.88E-15	Up	GCAUCAUCCAUCCUACCAUUC	21
tae-miR9669-5p	0	79	5.795663147	2.90E-14	5.36E-14	Up	UACUGUGGGCACUUAUUUGAC	21
tae-miR1120a	0	72	5.6618074	3.96E-13	7.15E-13	Up	ACAUUCUUAUAUUAUGAGACGGAG	24
tae-miR9653a-3p	0	68	5.57934524	1.79E-12	3.16E-12	Up	UUUGAGACUUUGGCCAUGGCC	21
tae-miR319	21	0	−6.900435024	8.05E-11	1.39E-10	Down	UUGGACUGAAGGGAGCUCCCU	21
tae-miR9775	0	55	5.273242112	2.64E-10	4.39E-10	Up	UGUGCGCAAUAAGAUUUUGCUA	22
tae-miR1125	0	55	5.273242112	2.64E-10	4.39E-10	Up	AACCAACGAGACCAACUGCGGCGG	24
tae-miR9657a-3p	0	52	5.192322117	8.51E-10	1.37E-09	Up	UGUGCUUCCUCGUCGAACGGU	21
tae-miR1117	0	47	5.04647125	6.10E-09	9.50E-09	Up	UAGUACCGGUUCGUGGCACGAACC	24
tae-miR1136	0	47	5.04647125	6.10E-09	9.50E-09	Up	UUGUCGCAGGUAUGGAUGUAUCUA	24
tae-miR398	19	5	−3.43411702	2.02E-08	3.05E-08	Down	UGUGUUCUCAGGUCGCCCCCG	21
tae-miR9782	0	38	4.739809912	2.24E-07	3.32E-07	Up	GUAUUAGGUUGGUCAAAUUGACGA	24
tae-miR6197-5p	0	37	4.701335764	3.36E-07	4.88E-07	Up	UCUGUAAACAAAUGUAGGACG	21
tae-miR1122b-3p	0	36	4.6618074	5.05E-07	7.18E-07	Up	AGACUUAUAUGUAGGAACGGA	21
tae-miR396-5p	0	34	4.57934524	1.14E-06	1.59E-06	Up	AACUGUGAACUCGCGGGGAUG	21
tae-miR5049-3p	0	31	4.446078709	3.91E-06	5.31E-06	Up	AAUAUGGAUCGGAGGGAGUAC	21
tae-miR9656-3p	0	31	4.446078709	3.91E-06	5.31E-06	Up	CUUCGAGACUCUGAACAGCGG	21
tae-miR9654b-3p	0	26	4.192322117	3.11E-05	4.11E-05	Up	UUCCGAAAGGCUUGAAGCGAAU	22
tae-miR2275-3p	0	23	4.015444355	0.00010918	0.000141743	Up	UUUGGUUUCCUCCAAUAUCUCG	22
tae-miR9661-5p	0	20	3.813810494	0.000387067	0.000493844	Up	UGAAGUAGAGCAGGGACCUCA	21

### Identification of novel miRNAs

**Figure 3:**
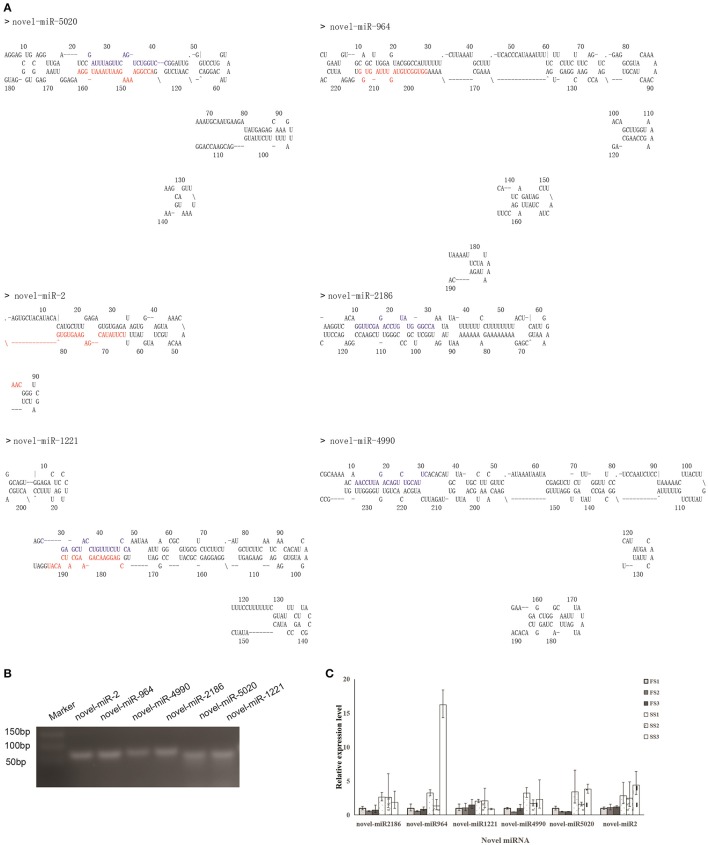
Novel miRNAs predicted and validated in this study. **(A)** Stem-loop structures of novel miRNA precursors (novel-miR-2, 1221, 964, 2186, 4990, and 5020) were constructed using MFOLD. Mature miRNA 5′ and 3′ sequences are highlighted in red and blue, respectively **(B)** Stem-loop RT-PCR analysis of the predicted novel miRNAs. Marker is a 50-bp DNA Ladder Marker. **(C)** qRT-PCR analysis of expression of the predicted novel miRNAs in different plants.

Novel miRNAs could be distinguished from other small RNAs by mapping to genomes of target species to predict secondary structures (Zuker, 2003; Yan et al., 2015). Through mapping onto their corresponding precursors, thousands of novel miRNAs were found, ranging from 917 to 7,762 in six libraries (6825 in SS1, 6700 in SS2, 2483 in SS3, 7762 in FS1, 6277 in FS2, and 917 in FS3) (Table 5); many of these miRNAs had low expression levels (Table S3). To validate the predicted novel miRNAs, 6 novel miRNAs including novel-miR-2, novel-miR-964, novel-miR-4990, novel-miR-2186, novel-miR-5020, and novel-miR-1221, were selected randomly for confirmation by stem-loop RT-PCR. DNA fragments of approximately 60 bp were amplified (Figure 3B), suggesting that these miRNAs were all expressed in the young panicles of BS366. Moreover, the folding structures for these novel miRNA precursors were predicted using MFOLD (http://mfold.rna.albany.edu/). Six structures of the novel miRNAs with perfect secondary structures are shown in Figure 3A. All of these six novel miRNAs were expressed in both fertile plants and sterile plants (Figure 3C). Furthermore, all of these novel miRNAs were up-regulated in sterile plants at the three developmental stages (Figure 3C). The novel miRNAs were named sequentially, according to the chromosome locations, and are described as novel-miR-number.

**Table 5:** Novel miRNAs prediction.

**Sample**	**Number of novel miRNA**	**Total expression of all novel miRNAs**
SS1	6,825	133,621
SS2	6,700	143,063
SS3	2,483	49,027
FS1	7,762	165,780
FS2	6,277	121,008
FS3	917	20,501

### miRNA target prediction and functional analysis

**Figure 4:**
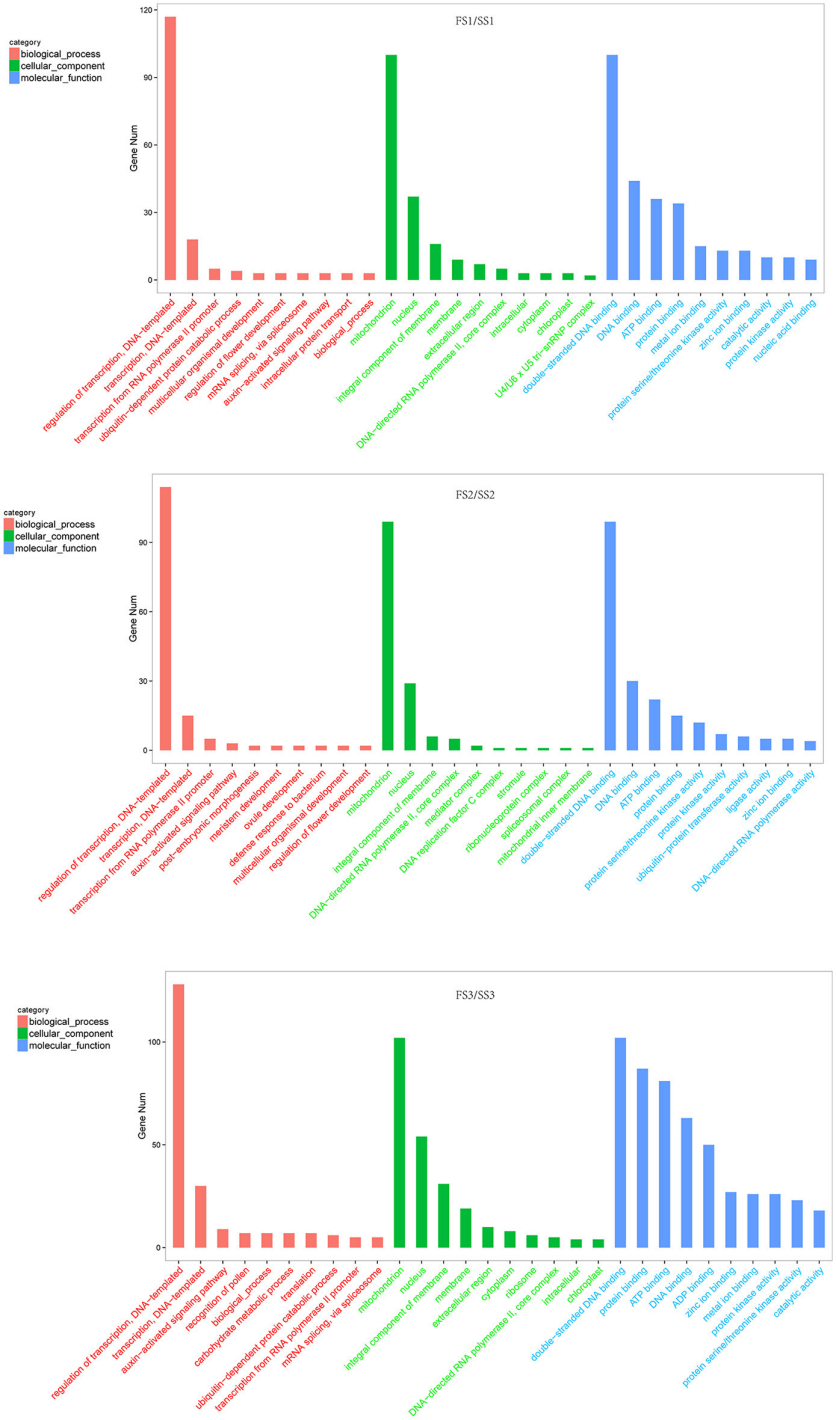
Gene ontology classification of differentially expressed targets of wheat miRNAs (fertile line/sterile line; FS/SS). In this ontology, “biological process,” “cellular component,” and “molecular function” are treated as independent attributes.

Identifying the candidate genes targeted by the miRNAs is crucial to understanding the biological functions of these miRNAs. To further investigate the regulatory effect of miRNAs, the sequences of all known and novel miRNAs were aligned to the wheat genome (downloaded from phytozome v11) to predict the potential targets genes (Allen et al., 2005). We found 3,286 targets for unique known miRNAs (Table S4-1) and 17,016 targets for novel miRNAs (Table S4-2). The targets of differentially expressed miRNAs of wheat were the focus of this study. These predicted targets of known miRNAs, such as miR156, miR159, miR164, miR1120, and miR167, are described as growth-regulating factors, MYB family transcription factors, F-box domain-containing proteins, MADS-box family proteins, and SBP-box gene family members (Table S5). These proteins are known to play various roles during plant growth and development processes. To more thoroughly investigate miRNA functions during male fertility transition in PTGMS wheat, gene ontology analysis of the predicted targets was performed. The most frequent term for “biological process” was “regulation of transcription” [such as the targets of tae-miR1120c-5p (Traes_4BS_35F8C45F6), tae-miR1130b-3p (Traes_2AL_EE1350B36) and tae-miR164 (Traes_2BL_6AEE8AC28)] followed by “transporter” and “auxin-activated signaling pathway” [such as the targets of tae-miR167a (Traes_2AL_A7941CB12)] in three comparison groups (FS1/SS1, FS2/SS2 and FS3/SS3) of targets for known miRNAs (Figure 4 and Table S6). In addition, the terms “regulation of flower development” [such as the targets of tae-miR167a (Traes_2DL_8434C0251), tae-miR1130b-3p (Traes_2DL_DA1A74C0D) and tae-miR156 (Traes_2BS_186EA570A)] and “recognition of pollen” [such as the targets of tae-miR1122b-3p (Traes_1DS_7868656E4.1) and tae-miR5049-3p (Traes_2AL_2A541092D.2)] were annotated in targets of differentially expressed miRNAs of FS1/SS1and FS3/SS3, respectively, but not in FS2/SS2. The most frequent “molecular function” term was “double−stranded DNA binding” and followed by “DNA binding,” “ATP binding,” and “protein binding” (Figure 4 and Table S7). The most frequent “cellular component” term was “mitochondrion.”

Furthermore, these targets were subjected to KEGG pathway analysis. Among these enriched pathways, “purine metabolism,” “spliceosome,” and “plant hormone signal transduction” had high frequencies for targets of known miRNAs (Figure S2). In addition, the “calcium signaling pathway” (ko04020) was enriched by KEGG pathway analysis (Table S6).

### Target identification of miRNAs by degradome sequencing

To confirm accuracy of the targets of miRNAs, two degradome libraries were generated from the spikelets of the PTGMS wheat line in different fertility condition (fertile and sterile). A total of 35,971,562 (98.16%) clean reads out of 36,647,546 raw reads and 26,622,338 (97.72%) clean reads out of 27,244,395 raw reads were produced in fertile line (FS) and sterile line (SS) libraries, respectively. Furthermore, 26,400,645 (73.89%) and 18,959,057 (71.21%) unique reads could be matched with the wheat genome in FS and SS libraries, respectively (Table 6). The FS and SS libraries had 1,276,084 (20.39%) and 7,912,816 (12.64%) specific raw reads, respectively, of which 9,636,103 (46.21%) and 6,323,897 (30.33%) were specific unique reads, respectively (Table 6). The reads that were mapped to wheat unigenes were subjected to further analysis.

**Table 6:** Statistics of degradome sequencing data of PTGMS line BS366 under different condition.

**Sample**	**Total reads**	**Low quality value**	**Incorrect size**	**Adaptors**	**Contain N base**	**Clean reads**	**Specific reads (total)**	**Specific reads (unique)**	**Match genome**
FS	36,647,546	29,235	540,616	1,137	104,996	35,971,562 (98.16%)	1,276,084(20.39%)	9,636,103(46.21%)	26,400,645(73.89%)
SS	27,244,395	12,972	531,812	708	76,565	26,622,338 (97.72%)	7,912,816(12.64%)	6,323,897 (30.33%)	18,959,057(71.21%)

*FS and SS indicated PTGMS plants in sterile (SS) and fertile condition (FS) of three stages, respectively*.

**Figure 5:**
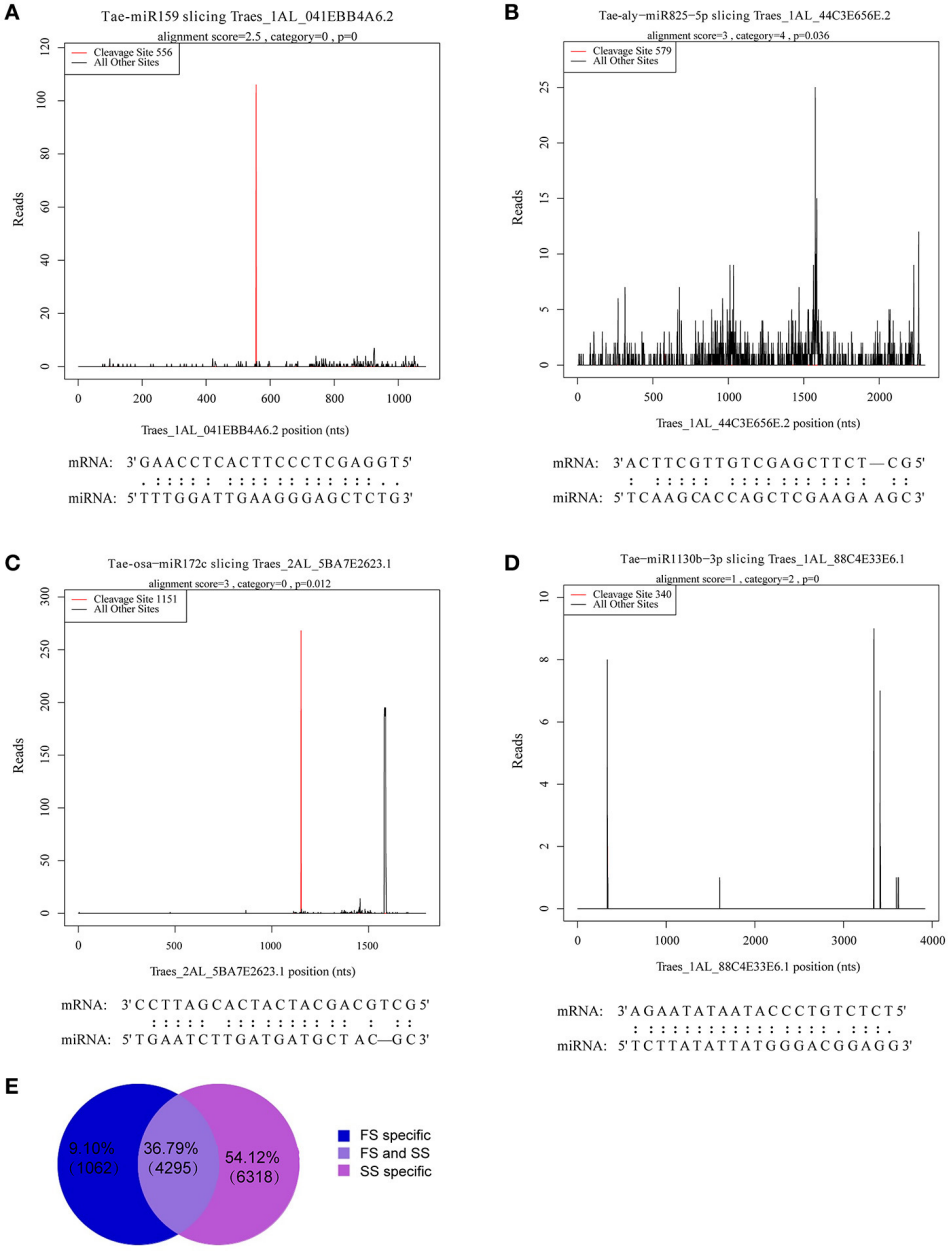
Analysis of known miRNA targets by degradome sequencing and statistics for annotated novel miRNAs targets. **(A–D)** Examples of target plots (t-plots) of known miRNA targets confirmed by degradome sequencing during the wheat male fertility transition. Alignment between representative miRNAs and their target transcripts are shown below the corresponding t-plot. **(A)** Targets of Tae-miR159, **(B)** targets of Tae-aly-miR825-5p, **(C)** targets of Tae-osa-miR172c, and **(D)** targets of Tae-miR1130b-3p. Red lines indicate the signature produced by miRNA-directed cleavage. **(E)** Statistics of annotated novel miRNAs targets.

A total of 5,357 targets in FS and 10,613 targets in SS were annotated. Among these targets, 1,062 (9.1%) and 6,316 (54.12%) were sample specific in FS and SS, respectively. A total of 5,357 targets in FS and 10,613 targets in SS were annotated (Figure 5E). The signature abundance of each target was plotted using absolute read number along the target transcripts (t-plot) (German et al., 2008). Four representative t-plots of selected targets are shown in Figures 5A–D. Degradome sequencing data suggested that targets of a miRNA family belong to the same gene family. Moreover, different genes belonging to the same gene family can also be degraded by different miRNA families. For example, GAMYB (TRAES3BF027700010CFD_t1) was degraded by miR159m-3p, miR159a, and miR159m-5p, suggesting that miRNA regulation is complex. In addition, a total of 899 targets of differentially expressed miRNAs between FS and SS were obtained and analyzed by GO annotations and KEGG pathway analysis to more thoroughly investigate miRNA functions during male fertility transition in PTGMS wheat (Table S7).

### Validation of the miRNA expression analysis and their targets by qPCR

**Figure 6:**
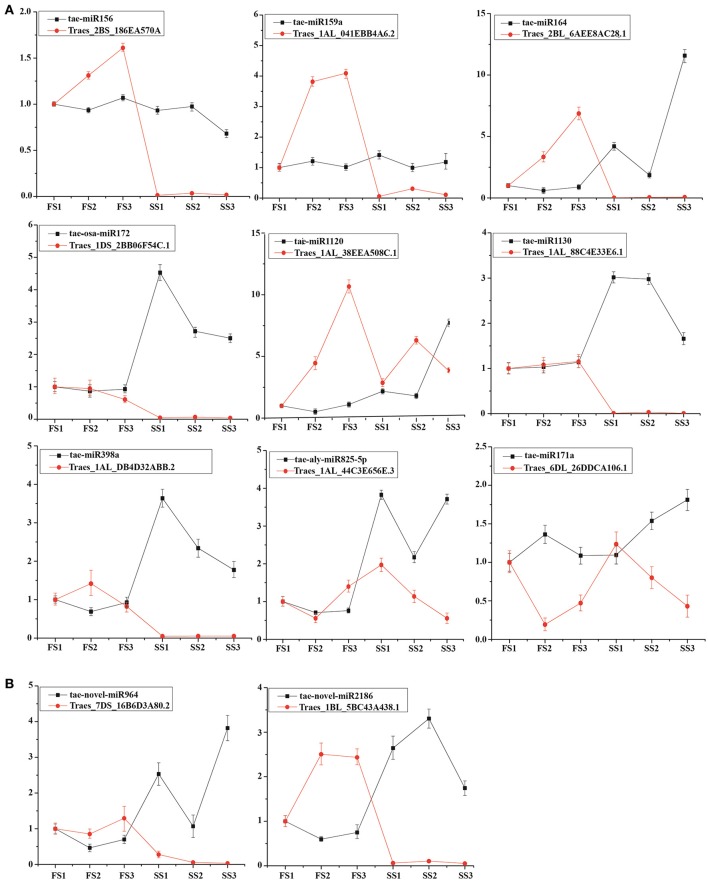
Expression profiling analysis of miRNAs and their target genes. Actin was used as a reference for the target genes. The error bars indicate the standard deviation of three replicates. **(A)** Known miRNAs and their target genes. **(B)** Novel miRNAs and their target genes.

To better understand the regulation of miRNA, we compared the differential expression of known and novel miRNAs between fertile and sterile plants at the same developmental stage. Usually, miRNAs play their roles by interacting with their targets. To examine the functional relationship between the targets and their corresponding miRNAs, 9 known miRNAs (tae-miRNA156, tae-miRNA171, tae-miRNA159, tae-miRNA172, tae-blo-miRNA398, tae-miRNA164, tae-miRNA825, miRNA1120, and miRNA1130) and 2 novel miRNAs (novel-miR-964 and novel-miR-2186) and expression of their targets were examined by qRT-PCR to analyze their principle regulation during male fertility transition (Figure 6). Tae-miR156 targets the SBP (SQUAMOSA-promoter binding protein) domain (Traes_2BS_186EA570A), miR164 targets the *CUP-SHAPED COTYLEDON* (*CUC*)/*NO APICAL MERISTEM* (*NAM*) gene family (Traes_2BL_6AEE8AC28.1), miR159 targets a myeloblastosis (*MYB*) transcription factor (Traes_1AL_041EBB4A6.2), tae-miR171a targets the *SCL* (*Scarecrow-like*) gene (Traes_6DL_26DDCA106.1), tae-aly-miR825-5p targets the *CaBP* gene (Ca^2+^ binding transmembrane protein) (Traes_1DL_F77022E34.2), tae-aly-miRNA398a-5p and tae-blo-miRNA398a-5p may target the *EXPB* gene (expansin precursor protein) (Traes_1AL_DB4D32ABB.2), tae-osa-miR172a targets the AP2-like factor (Traes_1DS_2BB06F54C.1), and tae-miR1130 targets *LRR* (leucine rich repeats) (Traes_1AL_88C4E33E6.1). Among these targets, the targets of miR156, miR164, and tae-miR171a were predicted, and the others were confirmed using degradation sequencing. These miRNAs with their targets were examined by qRT-PCR. It was found that the expression of most miRNAs was negatively correlated with their targets (Figure 6) between fertile plants (FS) and sterile plants (SS) during pollen development. In addition, two novel miRNAs were selected to verify the correlation. Both novel-miR-964 and novel-miR-2186 target the *pentatricopeptide repeat* (*PPR*) gene family (Traes_7DS_16B6D3A80.2 and Traes_1BL_5BC43A438.1, respectively). qRT-PCR showed that the expression of these two novel miRNAs is also negatively correlated with their targets (Figure 6B). These negative correlations suggest that miRNA-mediated mRNA silencing during pollen fertility transition period probably caused male sterility.

## Discussion

Recently, many studies have indicated that some miRNAs regulate anther development and have some relationship to male sterility (Tang Z. et al., 2012; Yan et al., 2015; Zhang et al., 2016). The relationship between CMS and miRNAs have been studied in some plants such as cotton (Wei et al., 2013), maize (Shen et al., 2011), and rice (Yan et al., 2015). In addition, very recent research proposed a possible model for the control exerted by miRNAs on signaling pathways during fertility transition in a rice PTGMS line (Zhang et al., 2016). In wheat, Tang Z. et al. (2012) analyzed the expression of miRNAs in TGMS wheat line when responsed to temperature (cold stress) to study the regulation of miRNAs (Tang Z. et al., 2012). However, the investigation of miRNAs in PTGMS wheat lines is very limited. Therefore, characterizing the role of miRNAs in PTGMS wheat lines would be extremely useful and could contribute to an improved understanding of the molecular functions of miRNAs during male fertility transition in the male sterile wheat lines used in two-line breeding systems. In this study, we examined miRNA profiles determined by high-throughput sequencing and qRT-PCR to investigate the expression of miRNAs in the PTGMS wheat line BS366 during pollen development (male fertility transition). In order to construct miRNA-gene regulatory network to illustrate the role of miRNA in male fertility transition in the male sterile wheat line, we not only investigated the regulation of miRNAs which responsed temperature during pollen development, but also analyzed the contribution of photoperiod for pollen development. These analyses showed that miRNAs interacting with their targets may play an important role in pollen development. In the present study, a total of 58 wheat-specific known miRNAs were identified in the spike of BS366 as differentially expressed in sterile and fertile conditions (Table 4). Targets of these differentially expressed miRNAs include MYB family protein, kinases, RNA-binding proteins, PRR-containing proteins, and stress-resistance related proteins (Tables S5, S6), implying that these proteins may play a particular role in pollen development.

In plants, it is well known that miR164 targets the *NAM* gene family [*CUC1* (*CUP-SHAPED COTYLEDON1*) and *CUC2* (*CUP-SHAPED COTYLEDON2*)] which are a type of plant-specific transcription factor; they have been shown to participate in various physiological and biochemical processes during plant development and in response to biotic/abiotic stresses (Kikuchi et al., 2000; Fujita et al., 2004; Hu et al., 2006; Takasaki et al., 2010; Tran et al., 2010; Nuruzzaman et al., 2012; Tang Y. et al., 2012). To date, several *NAM* genes have been cloned and characterized in wheat, and most can be induced by biotic/abiotic stresses (Takasaki et al., 2010; Xia et al., 2010; Tang Y. et al., 2012). miR164 is also involved in age-dependent cell death in *Arabidopsis* leaves by cleaving ORE1, which is another *NAM* transcription factor (Kim et al., 2009). In addition, *mir164-CUC1* and *mir164-CUC2* plants both exhibit leaf shape and polarity defects, extra petals, missing sepals, and reduced fertility (Laufs et al., 2004; Mallory et al., 2004). In addition, miR164 was up-regulated under UV-B radiation treatment in maize, suggesting that the expression of miR164 might be regulated by light (Casati, 2013). In the present study, tae-miR164 was found to be down-regulated in the pollen at later developmental stages (SS2 and SS3) in sterile plants (Table 4) but not in SS1/FS1, and this finding was verified by qRT-PCR (Figure 6A), indicating that miR164 may participate in fertility regulation during pollen development. The expression of target Traes_2BL_6AEE8AC28.1, which was annotated as the *CUP-SHAPED COTYLEDON (CUC)/NO APICAL MERISTEM* (*NAM*) gene family, was examined by qRT-PCR. This target was down-regulated in the sterile group, especially in later anther developmental stages (SS2 and SS3) (Figure 6A) demonstrating that the target was silenced by miRNA.

Recently, it has been reported that miR156 (Schwab et al., 2005), miR159 (Achard et al., 2004), and miR172 (Aukerman and Sakai, 2003; Chen, 2004 are involved in control of flowering time. miR156, which targets *SPL* (*SQUAMOSA PROMOTER BINDING PROTEIN-LIKE*) transcription factors, promotes flowering and plays important regulatory roles throughout the growth and development stages (Cardon et al., 1997; Schmid et al., 2003). Indeed, recent studies have shown that vegetative phase changes in *Arabidopsis* could be regulated by miR156 which promotes the expression of the juvenile phase and represses the expression of the adult phase (Yang et al., 2011). miR172 promotes flowering primarily by repressing a set of *AP2-like* genes (*TOE1, TOE2*, and *TOE3*) (Aukerman and Sakai, 2003), whereas miR172-overexpressing plants exhibit early flowering under both long days (LDs) and short days (SDs). In addition, it has been reported that the expression of miR172 could be mediated by *Cryptochrome 1* (*CRY1*) and *Cryptochrome 2* (*CRY2*) after blue light stimulation involving *GIGANTEA* (*GI*)-mediated photoperiodic flowering (Jung et al., 2007; Zhou et al., 2016). In the present study, the target of miR172 (Traes_1DS_2BB06F54C.1), which was confirmed by degradome sequencing, was negatively correlated with miR172 and down-regulated in SS, whereas miR172 was significantly up-regulated. miR156 has been known to play a crucial role in the development of sporogenic tissues with its targets in rice (Yamaguchi et al., 2009) and *Arabidopsis* (Xing et al., 2010). The targets of miR156 (Traes_2BL_186EA570A), annotated as the *SBP* family (for *SQUAMOSA-PROMOTER BINDING PROTEIN*) which is a sequence specific DNA-binding domain found in plant proteins, was nearly silenced in SS. miR159 targets *MYB* transcription factors, playing important roles in flower development (Achard et al., 2004; Jones-Rhoades et al., 2006). In rice, mutations of *OsGAMYB* resulted in defects in the anthers and pollen development (Kaneko et al., 2004). Moreover, studies have shown that the abundance of miR159 is directly related to fertility in *Arabidopsis* (Achard et al., 2004) and rice (Tsuji et al., 2006). Furthermore, overproduction of miR159 resulted in late flowering specifically under SDs. In this study, the target of tae-miR159/tae-miR319 (Traes_1AL_041EBB4A6.2) was down-regulated significantly suggesting that tae-miR159 might be involved in regulation of anther development.

We also found that there was differential expression of the calmodulin-binding protein (Table S7). As an important second messenger, calcium ions (Ca^2+^) participate in a number of physiological responses in both animal and plant cells. The production and transduction of calcium signals are realized through the change in calcium distribution and concentration in cytoplasm and organelles (Dodd et al., 2010). Studies demonstrated that Ca^2+^ signals contribute to cryptochrome (*CRY*) and phytochrome (*PHY*) signaling (Spalding, 2000; Guo et al., 2001). In addition, calcium concentrations that are inappropriately located may cause pollen development defects, resulting in male sterility in PGMS (photoperiod-sensitive genic male-sterile) rice (Tian et al., 1998). There are significant differences in calcium distribution between fertile and sterile anthers. In rice, some studies indicated that osa-miR1432 and osa-miR812d may be involved in Ca^2+^-mediated signaling pathways through interaction with their target genes (Yan et al., 2015). Osa-miR5976 showed up-regulated expression in rice sterile lines (Zhang et al., 2016). In our study, tae-aly-miR825-5p was verified as targeting calmodulin binding protein by degradome sequencing (Traes_1BL_F0F995FE3.1), and expression of tae-aly-miR825-5p was up regulated in sterile lines (Table 4 and Table S6). Transcripts of Traes_1BL_F0F995FE3.1 were positively correlated with the expression of tae-aly-miR825-5p (Figure 6A). Therefore, the Ca^2+^ signal pathway also may be regulated by environmental factors and miRNA, causing male sterility in wheat.

There are many studies demonstrating that miR167 with its targets plays a crucial role in floral organ development. Overexpression of miR167 leads to *Arabidopsis* male sterility (Siré et al., 2009). Mutation of auxin response factor genes *ARF6* and *ARF8* (targets of miR167) could cause flower organ developmental defects (Nagpal et al., 2005; Peng et al., 2006). The targets of tae-miR1120 and tae-miR1130 were annotated as the *lipid phosphate phosphatase* (*LPP*) gene, showing differential expression between FS and SS (Table S5). The *Arabidopsis* miR171 family with their targets are involved in shoot branching (Wang et al., 2010; Manavella et al., 2013) and *gibberellin* (*GA*)*-DELLA* signaling-mediated chlorophyll biosynthesis (Ma et al., 2014). Moreover, miR171 target protein LiSCL (*L. longiflorum* Scarecrow-like), also called GRAS, is expressed specifically at the premeiotic phase within anthers (Morohashi et al., 2003). Recently, studies reported that miR171 and miR167 showed diurnal oscillation expression during the day: up-regulated in the light and down-regulated in darkness (Siré et al., 2009). Another study showed that blue light alters miR167 expression and microRNA-targeted auxin response factor genes in *Arabidopsis thaliana* suggesting that miR171 and miR167 may be regulated by light or the circadian clock. In this study, tae-miR171a and tae-miR167a show differential expression between FS and SS at different developmental stages (Table 4), as well as their targets (Traes_6DL_26DDCA106.1 for tae-miR171a; Traes_2AL_A7941CB12.2 and Traes_2BL_B2D711406.2 for tae-miR167a) (Table S5). Furthermore, there are consistent negative relationships between expression of tae-miR171a and its target in different developmental stages, suggesting that tae-miR171a is a negative regulator in post-transcriptional gene silencing through base pairing with target mRNAs, which leads to mRNA cleavage or translational repression.

There are other miRNAs involved in male sterility. It has been reported that miR398 targets *Cu/Zn superoxide dismutase* genes, affecting oxidative stress tolerance (Dugas and Bartel, 2008; Pashkovskii et al., 2010; Zhu et al., 2011). In the present study, miR398, targeting the *EXPB* gene, was identified by degradome sequencing analysis. It has been reported that *EXPB* genes may be related to pollen germination ability at the young microspore stage in rice (Hayashi et al., 2009). In this study, tae-aly-miRNA398a-5p/tae-blo-miRNA398a-5p may target the *EXPB* gene family, being up-regulated in sterile plants (Table 2). Moreover, high expression of tae-aly-miRNA398a-5p/tae-blo-miRNA398a-5p inhibited target gene (Traes_1AL_DB4D32ABB.2) expression (Figure 6A).

The *pentatricopeptide repeat* (*PPR*) genes code for PPR proteins (PPRPs) that are putative RNA-binding proteins (Lurin et al., 2004). Studies have indicated that PPRPs are involved in plant development (Cushing et al., 2005; Prasad et al., 2005), are restorers of CMS (Bentolila et al., 2002; Brown et al., 2003; Koizuka et al., 2003; Akagi et al., 2004), process chloroplast and mitochondrial RNA (Meierhoff et al., 2003; Nakamura et al., 2003; Lurin et al., 2004), and RNA editing in chloroplasts (Miyamoto et al., 2004; Kotera et al., 2005). In the present study, it was found that two novel miRNAs, tae-novel-miR964 and tae-novel-miR2186, exhibited differential expression among FS1/SS1, FS2/SS2, and FS3/SS3 (Table S3). In addition, both their target genes (Traes_7DS_16B6D3A80.2 and Traes_1BL_5BC43A438.1, respectively) were annotated as *PPR* family genes (Table S7). These two miRNAs were up-regulated in three developmental stages of sterile plants, whereas their targets were down-regulated (Figure 6B), suggesting that these two novel-miRNAs play an important role in pollen development by silencing the expression of their targets.

**Figure 7:**
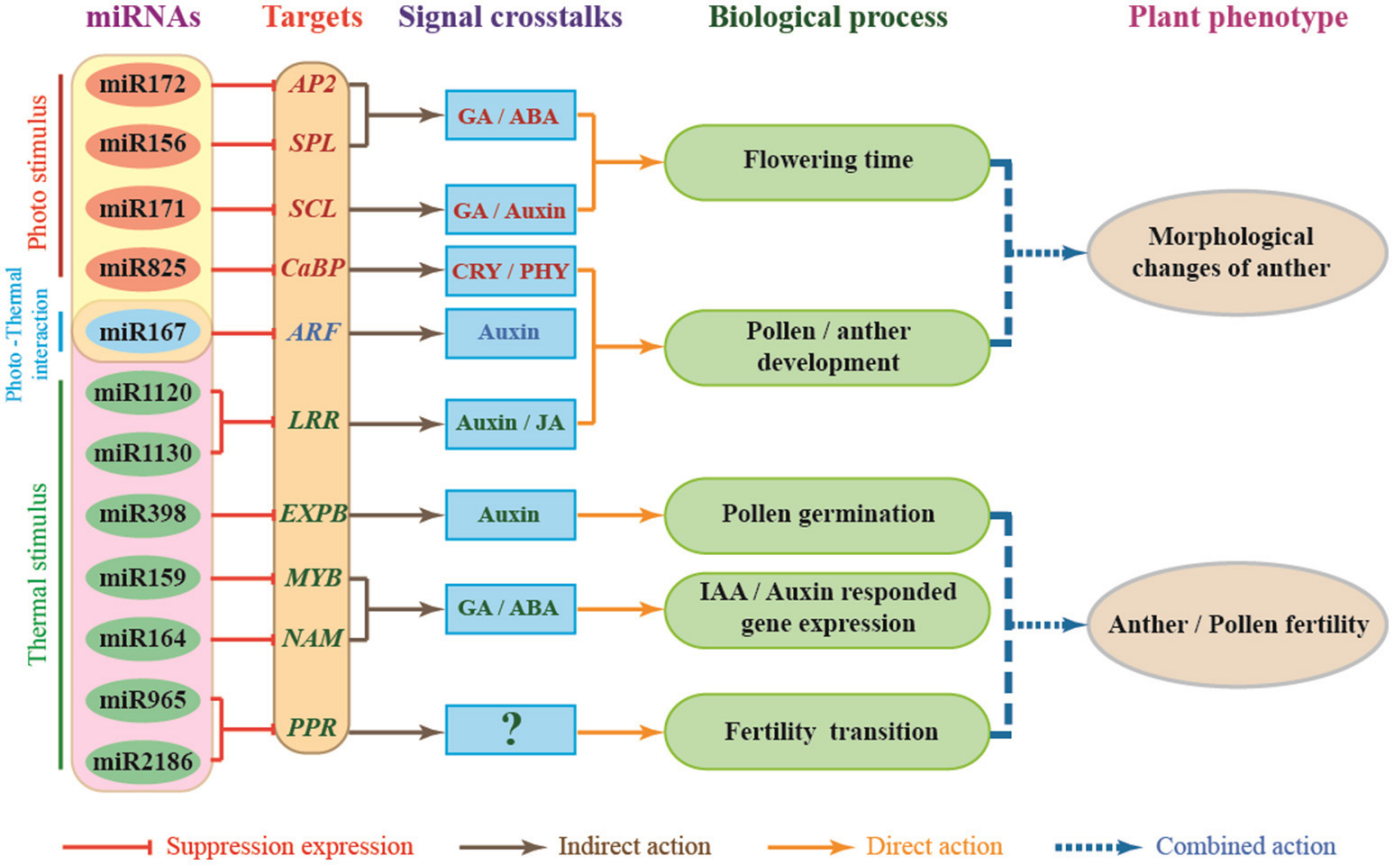
Proposed miRNA-dependent regulatory pathway that participate in wheat male fertility transition. *CaBP*, Ca^2+^ binding protein gene; *AP2, APETALA2* gene; *SPL, TaSPL-SBP-box* gene family member; *SCL*, Scarecrow-like gene family; *ARF*, auxin response factor; *LRR*, leucine rich repeat gene; *EXPB*, β-expansins gene; *NAM, NO APICAL MERISTEM* gene family; *PPR*, pentatricopeptide repeat protein; *MYB*, myeloblastosis family transcription factor.

Lots of target genes of miRNAs have been reported in the environmental stress response and are directly involved in some hormone-related signaling pathways that regulate plant development (Shinozaki, 2007). As *AP2* transcription factors, *AREB/ABFs* are involved in the ABA-dependent signal transduction pathway (Yamaguchishinozaki and Shinozaki, 1994, 2005; Uno et al., 2000; Kang et al., 2002; Fujita and Yamaguchi-Shinozaki, 2005; Furihata et al., 2006). *MYB2* function in ABA- and JA-inducible gene expressions (Abe et al., 2003). The *RD26 NAC* transcription factor is involved in ABA- and JA-responsive gene expression in stress responses (Abe et al., 2003; Tran et al., 2004; Tang Y. et al., 2012; Wang et al., 2016). Recently, it has been reported that up- or down-regulation of *JAZ* (*JASMONATE-ZIM DOMAIN*) genes might be associated with the abnormal anther dehiscence in TGMS wheat line (Wang et al., 2017). Based on the above analysis, we developed a model to try to describe miRNAs-mediated gene regulation networks and the signaling pathway involved in male sterility of PTGMS wheat line BS366 (Figure 7). As shown in Figure 7, external environment signals, including light and temperature, affect wheat through regulation of miRNAs and their targets. In this model, miR825, miR172, miR156, and miR171 are mainly regulated by light, whereas miR1130/miR1120, miR398, miR159, miR164, and two novel-miRNAs (novel-miR964 and novel-miR2186) may be regulated by light. Moreover, miR167 is regulated by both light and temperature. miR172, miR156, and miR171 interact with their respective target genes (*AP2, SPL*, and *SCL*), participating in the GA/abscisic acid (ABA) signaling pathway and GA/auxin signaling pathway to regulate flowering time; miR825, miR167, and miR1120/miR1130 interact with their respective target genes (*CaBP, ARF*, and *LRR*), participating in the CRY/PHY signaling pathway, auxin signaling pathway and JA signaling pathway, to modulate pollen development. These interactions result in morphological changes of the wheat pollen. In contrast, the interactions of miR398, miR159, miR164, and novel-miR964/novel-miR2186 with their corresponding target genes (*EXPB, MYB, NAM*, and *PPR*) participate in the auxin signaling pathway and the GA/ABA signaling pathway to modulate pollen germination, auxin/IAA responded gene expression and male fertility transition to result in defects of pollen fertility. Photo-thermosensitive genic male sterility in wheat might be the result of the combination of the regulatory interactions described above. It is therefore clear that miRNA-mediated photo-thermosensitive genic male sterility in wheat is a very complicated network regulation system involving the expression of genes, proteins, and regulation of hormone signaling. This study may offer important clues for understanding the different ways gene expression is regulated during male fertility transition by functional description and target analysis of miRNAs in wheat. These miRNA profiles also provide an important theoretical foundation for two-line hybrid wheat molecular breeding and may produce insights for further investigations of PTGMS wheat lines.

## Conclusions

In this study, data from small RNA-seq and degradome-seq were analyzed to examine the regulation of miRNAs of the PTGMS wheat line BS366 during male fertility transition. The expression and correlation of 11 selected flowering and/or male sterility-related miRNAs/targets were detected by qRT-PCR in different fertility conditions during male fertility transition. Most miRNAs/targets had negatively correlated expression and may participate in the signaling pathway during male fertility transition. In addition, a miRNA-mediated regulation mechanism during PTGMS in the wheat line BS366 was developed via more extensive function analysis. A number of newly identified miRNAs were also detected. Our data could be used as a benchmark for future studies of the molecular mechanisms of PTGMS in other crops.

## Author contributions

JB, YW, LZ, and CZ designed research scheme, JB, YW, PW, WD, SY, and HS performed experiments, JB, YW, PW, JM, and WD analyzed data, JB, YW, and PW wrote the manuscript, JB, YW, PW, LZ, CZ, FZ, NW, GY, and SY revised it. All authors read and approved the final manuscript.

### Conflict of interest statement

The authors declare that the research was conducted in the absence of any commercial or financial relationships that could be construed as a potential conflict of interest.

## Supplementary material

Figure S1 The first nucleotides analysis of the differentially expressed 21nt miRNAs showed that the bases rates of U, C, A and G.

Figure S2 The top 20 enriched KEGG pathways of predicted target genes of the differentially expressed targets between developmental stages of fertile (FS) and sterile (SS) lines. Comparison between **(A)** FS1 and SS1, **(B)** FS2 and SS2, and **(C)** FS3 and SS3 libraries. The ordinate indicates the KEGG pathway which differentially expressed targets participated. The abscissa indicates enrichment score. The size of color points indicate the number of each target in KEGG pathway.

Table S1 Primers used in this study.

Table S2 Expression of wheat-specific miRNA in six library.

Table S3 Comparison of the expression levels for the novel miRNAs between SS and FS among different decelopment stages.

Table S4 Summary of miRNA target prediction.

Table S5 Target prediction of known miRNA having differential expression.

Table S6 List of KEGG enrichment of predicted targets.

Table S7 miRNA targets identified by degradome analysis during anther development stages.
