# Supplementary figures and images for: Uncovering Male Fertility Transition Responsive miRNA in a Wheat Photo-Thermosensitive Genic Male Sterile Line by Deep Sequencing and Degradome Analysis
Source: Front Plant Sci. 2017 Aug 8;8:1370. doi: 10.3389/fpls.2017.01370 (PMC5550412; doi:10.3389/fpls.2017.01370)

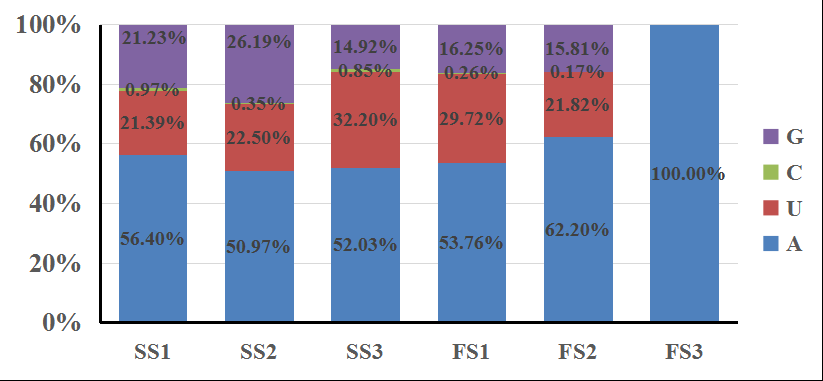

Supplement: Figure S1 — The first nucleotides analysis of the differentially expressed 21nt miRNAs showed that the bases rates of U, C, A and G. [file Image1.tif]

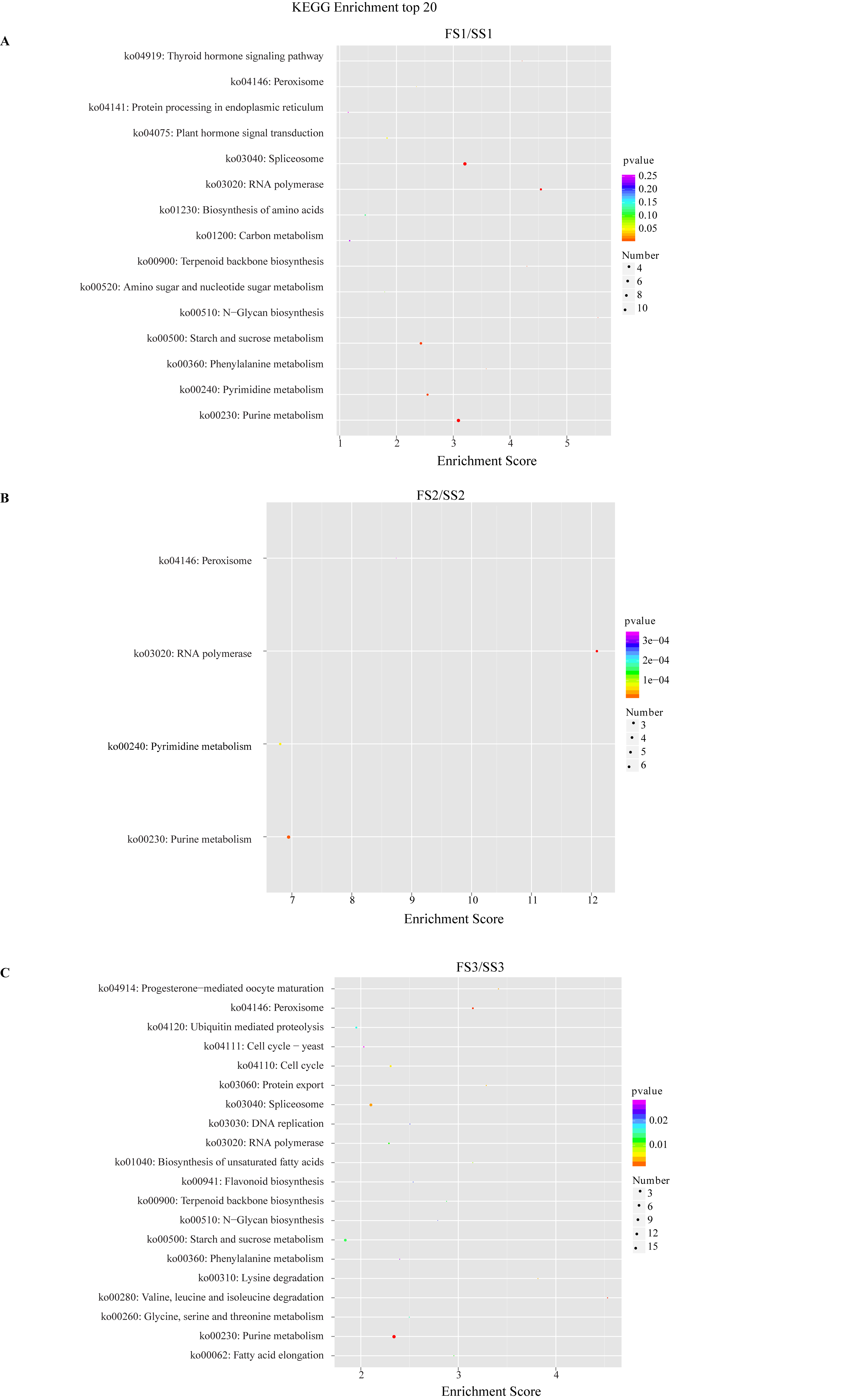

Supplement: Figure S2 — The top 20 enriched KEGG pathways of predicted target genes of the differentially expressed targets between developmental stages of fertile (FS) and sterile (SS) lines. Comparison between (A) FS1 and SS1, (B) FS2 and SS2, and (C) FS3 and SS3 libraries. The ordinate indicates the KEGG pathway which differentially expressed targets participated. The abscissa indicates enrichment score. The size of color points indicate the number of each target in KEGG pathway. [file Image2.tif]
